# Expert Consensus Develops Multidisciplinary Pathway for Cancer Pain Management in Italy: A Delphi Study

**DOI:** 10.3390/curroncol33090507

**Published:** 2026-08-26

**Authors:** Francesco Cellini, Leonardo Consoletti, Massimo Di Maio, Diego Maria Michele Fornasari, Gianpaolo Fortini, Marta Gentili, Marco Krengli, Ernesto Maranzano, Silvia Natoli, on behalf of the Cancer Pain Management in Italy Working Group

**Affiliations:** 1 Department of Diagnostic Imaging, Oncologic Radiotherapy and Hematology, Universita Cattolica del Sacro Cuore, 00168 Rome, Italy; f.cellinimd@gmail.com; 2 Department of Diagnostic Imaging, Oncologic Radiotherapy and Hematology, Fondazione Policlinico Universitario “A. Gemelli” IRCCS, 00168 Rome, Italy; 3 Pain Management Unit, “Policlinico Riuniti” University Hospital of Foggia, 71122 Foggia, Italy; leonardoconsoletti@libero.it; 4 Federdolore–Italian Society of Pain Clinicians (SICD); 5 Department of Oncology, AOU Citta della Salute e della Scienza di Torino, University of Turin, 10124 Turin, Italy; massimo.dimaio@unito.it; 6 Italian Association of Medical Oncology (AIOM), 20133 Milan, Italy; 7 Department of Medical Biotechnology and Translational Medicine, Università degli Studi di Milano, 20122 Milan, Italy; diego.fornasari@unimi.it; 8 The Italian Association for the Study of Pain, 00193 Rome, Italy; 9 Integrated Palliative Care Unit, ASST Sette Laghi, 21100 Varese, Italy; gianpaolo.fortini@asst-settelaghi.it; 10 The Italian Society of Palliative Care (SICP), 20124 Milan, Italy; 11 Palliative Care and Pain Therapy, DG Welfare, Lombardy Region, 20100 Milan, Italy; 12 Fondazione Nora e Alberto Gentili ETS; 13 Department of Surgical, Oncological and Gastroenterological Sciences (DISCOG), University of Padua, 35122 Padua, Italy; marco.krengli@unipd.it; 14 Division of Radiotherapy, Veneto Institute of Oncology IOV IRCCS, 35122 Padua, Italy; 15 Department of Radiation Oncology, University of Perugia, 06123 Perugia, Italy; ernesto.maranzano@libero.it; 16 Department of Clinical-Surgical, Diagnostic and Pediatric Sciences, University of Pavia, 27100 Pavia, Italy; silvia.natoli@unipv.it; 17 Pain Unit, Fondazione IRCCS Policlinico San Matteo, 27100 Pavia, Italy; 18 Italian Society of Anesthesiology, Analgesia, Resuscitation and Intensive Care (SIAARTI)—Section of Pain Medicine and Palliative Care, 00184 Rome, Italy

**Keywords:** cancer pain management, clinical pathway, Delphi consensus, multidisciplinary approach, opioid prescribing, pain assessment, palliative care

## Abstract

Background: Cancer pain remains highly prevalent and undertreated despite established guidelines. In Italy, Law 38/2010 mandates systematic pain assessment, yet only 26% of clinicians routinely evaluate pain at each clinical visit, and fewer than one-quarter have received formal training in pain medicine or palliative care. A national multidisciplinary roundtable, convened in Rome in March 2025, formally identified four systemic gaps—insufficient education, fragmented care pathways, unclear professional roles, and challenges in implementing shared diagnostic and therapeutic pathways—and planned the development of a structured Delphi consensus. Methods: A Delphi consensus process was undertaken in accordance with CREDES guidelines. The Steering Committee, comprising representatives of six Italian scientific societies (AIRO, AIOM, AISD, Federdolore-SICD, SICP, ACD-SIAARTI) and a patient advocacy group (Fondazione Nora e Alberto Gentili), developed 15 clinical statements addressing pain assessment, management, referral criteria, monitoring, and documentation over five online meetings held between March and September 2025. Sixty-six Italian clinicians from various specialties were invited to participate; the survey was open from 1 October to 31 December 2025, with reminders every 10 days. Consensus was defined as ≥75% agreement (scoring 4 or 5 on a 5-point Likert scale). Results: Fifty-six clinicians completed the survey (response rate: 84.8%), representing medical oncology, radiation oncology, pain therapy, and palliative care specialties; individual statements were rated by 53–54 panelists, as skipping single items was permitted. All statements reached consensus in the first round (77.8–100%), precluding the need for a second voting round. Panelists’ qualitative comments informed minor wording refinements; substantial content was unchanged. Conclusions: The Delphi process produced a validated, multidisciplinary clinical pathway for cancer pain management in the Italian National Health System (NHS). The pathway establishes structured roles for the clinical reference physician and specialist consultants, objective decision thresholds for analgesic titration and referral, and minimum requirements for standardized pain documentation. These consensus-based statements provide actionable clinical guidance that may help address analgesic undertreatment and support the implementation of Law 38/2010 across Italian oncology centers.

## Simple Summary

Cancer pain is common and can greatly affect quality of life, yet it is still not always assessed and treated consistently. In Italy, national legislation requires regular pain assessment, but differences in clinical practice and organization remain. This study brought together experts from several medical specialties to agree on a practical, shared approach to cancer pain management. Using a structured consensus process, the experts evaluated 15 statements covering pain assessment, treatment, early follow-up, referral to specialist care, and standardized documentation. All statements reached the predefined level of agreement. The resulting pathway clarifies who should assess and initially manage pain, when treatment response should be checked, and when other specialists should become involved. This shared approach may help reduce delays and undertreatment, improve coordination among healthcare professionals, and support more consistent cancer pain care across Italian oncology services.

## 1. Introduction

Pain is one of the most frequent and debilitating symptoms in patients with cancer. Landmark epidemiological studies estimate an overall prevalence of approximately 44.5%, rising to more than 50% in patients undergoing active treatment and exceeding 65% in patients with advanced or metastatic disease [1,2]. Moderate-to-severe pain affects around one third of patients, with profound consequences for quality of life, psychological well-being, and functional autonomy [1]. Despite decades of research and the availability of established guidelines, the problem of analgesic undertreatment remains unresolved: the Pain Management Index (PMI) has documented a portion of inadequately treated patients that, while slowly declining from 43% in the 1994–2007 period, still stood at approximately 40% between 2014 and 2020 [3,4,5].

The burden is global, and so is the treatment gap. The Lancet Commission on Palliative Care and Pain Relief estimated that tens of millions of people experience serious health-related suffering each year without access to adequate pain relief, with the overwhelming majority living in low- and middle-income countries, and identified the maldistribution of opioid analgesics as one of the most extreme inequities in global health [6]. The World Health Organization has responded with dedicated guidance on the pharmacological and radiotherapeutic management of cancer pain, which reaffirms that the objective of treatment is to reduce pain to a level compatible with a quality of life acceptable to the patient, and that balanced opioid policy is a precondition for achieving it [7]. Even in high-income systems with unrestricted opioid availability, however, the translation of these recommendations into routine oncological practice remains incomplete: palliative care is still frequently activated late in the disease trajectory, and the organizational arrangements that determine who assesses pain, and when, are rarely specified by clinical guidelines [6,7].

The Italian landscape presents distinctive challenges. Although Law 38/2010 introduced a statutory requirement for systematic pain assessment and documentation across all healthcare settings, its implementation can be seen as still suboptimal. Recent national surveys indicate that only 26% of clinicians routinely assess pain at every visit, only 17% monitor it daily during hospitalization, and merely 23.5% have completed formal training in pain medicine or palliative care [8,9,10]. Underutilization of validated assessment tools (Numeric Rating Scale [NRS], Edmonton Symptom Assessment System [ESAS], Douleur Neuropathique en 4 questions [DN4]), suboptimal analgesic sequencing and modest use of opioids remain widespread [4,11,12]. These clinical deficiencies are compounded by marked regional heterogeneity in infrastructure and prescribing policies, fragmented care pathways, and limited interdisciplinary integration—all of which contribute to pain management that is frequently delayed or inadequate [8].

To address these gaps, a national roundtable was convened in Rome on 28 March 2025, bringing together representatives of the major Italian scientific societies involved in cancer pain management—that is, the national societies of radiation oncology (AIRO), medical oncology (AIOM), the study of pain (AISD), pain clinicians (Federdolore–SICD), palliative care (SICP), and the pain and palliative care section of the society of anaesthesia and intensive care (ACD–SIAARTI)—and a patient advocacy group (Fondazione Nora e Alberto Gentili, Nora and Alberto Gentili Foundation). The roundtable identified four critical areas: insufficient education and training, fragmented care pathways, undefined professional roles, and challenges in implementing shared diagnostic and therapeutic care pathways (Percorsi Diagnostico-Terapeutici Assistenziali, PDTAs). As a result, the roundtable formally mandated the development of a structured Delphi process to translate the proposals into shared, actionable clinical recommendations [8].

The Delphi methodology is a structured, iterative technique for aggregating expert judgement in domains where the empirical evidence is incomplete, or where it does not translate directly into operational decisions. Four features define it: successive rounds of questioning; anonymity of individual responses; controlled feedback between rounds; and quantitative aggregation of the panel’s judgements against a threshold specified in advance [13,14]. Anonymity is a methodological requirement rather than an administrative convenience—it prevents individual opinions from being weighted by seniority, institutional affiliation, or the group dynamics that characterize face-to-face consensus meetings, and allows panelists to withhold agreement without social cost [13]. The threshold applied here is likewise not arbitrary: a systematic review of 100 Delphi studies in health research found percentage agreement to be the most frequent definition of consensus, with a median threshold of 75% [14]. The method is well established in cancer pain management and palliative care [13], and several prior initiatives have demonstrated its applicability in this field, from the development of multidimensional needs assessment instruments [15,16] to the validation of a standardized form for pain documentation in Italian oncology practice, developed through a Delphi process involving the directors of 18 medical oncology specialty schools [17].

The primary objective of the present study was to achieve a multidisciplinary consensus on a shared clinical pathway for cancer pain management in the Italian oncology setting, including the definition of criteria and timelines for interdisciplinary referral.

## 2. Methods

### 2.1. Study Design

The present work is a structured expert consensus process based on the Delphi technique. It is not an interventional or observational clinical study, and the term “study” is used throughout in the methodological sense in which it is applied to Delphi exercises by the CREDES recommendations. It is also distinct from a survey: the items submitted to the panel were not exploratory questions about current practice, but candidate normative statements formulated a priori by a Steering Committee and rated against a consensus threshold defined in advance, with provision for reformulation and re-voting of any statement failing to reach it. The Delphi technique was selected for its established suitability in developing clinical guidance within oncology and palliative care settings [13]. The process was conducted and reported in accordance with the CREDES (Conducting and REporting DElphi Studies) recommendations [13] and with the methodological reporting criteria proposed by Diamond et al. [14].

### 2.2. Statement Development

Fifteen clinical statements were developed by the Steering Committee between 28 March and 30 September 2025, over the course of five online meetings conducted as open, unstructured discussions, drawing on current evidence and the four thematic gaps identified at the 2025 national roundtable [8]: interdisciplinary role definition, pain assessment standards, standardized clinical documentation, and referral criteria and monitoring timelines. The statements spanned the domains of pain assessment, pharmacological and non-pharmacological management, specialist referral, and clinical documentation.

The development process proceeded in three stages. First, the four thematic gaps were adopted as the domain structure of the statement set. Those gaps had themselves been identified by consensus of the roundtable participants, on the basis of their clinical experience, of the national survey data available at the time [9,10], and of the documented shortfall in the implementation of Law 38/2010; the proceedings of that meeting, including the derivation of the four gaps, have been published separately [8]. Second, a literature search was performed to inform the content of each domain. This search was not a systematic review. PubMed was interrogated without date restriction, using terms relating to cancer pain assessment, opioid titration and re-modulation, multidisciplinary and specialist referral, and pain documentation in the clinical record; the retrieved records were supplemented by the personal literature collections of Steering Committee members. Publications were selected by the Committee on the grounds of relevance rather than against pre-specified eligibility criteria, and no formal grading of the level or quality of evidence was applied to individual statements. Third, candidate statements were drafted, discussed and reformulated across successive meetings until every member of the Steering Committee agreed that each statement was clinically pertinent, unambiguous in wording, and applicable within the organizational reality of the Italian National Health System. All members reviewed the final wording for clarity and face validity before dissemination. No pilot administration to an external sample of clinicians was undertaken prior to the voting round.

Where a statement embedded a quantitative threshold, that threshold was taken from published sources rather than generated de novo. In particular, the ≥30% reduction in pain intensity used in Statements 5 and 7 corresponds to the clinically important difference established for the 11-point NRS in pooled analyses of chronic pain trials and adopted in international guidance on cancer pain management [18,19]; the 3–7 day and 7-day intervals in Statements 4, 5 and 8 reflect the monitoring timelines proposed in the roundtable document [8].

### 2.3. Steering Committee and Panel Composition

The process was overseen by a multisociety Steering Committee comprising representatives from AIRO, AIOM, AISD, Federdolore-SICD, SICP, and ACD-SIAARTI. Committee members were not self-selected. Each participating society formally designated its own representative or representatives, on the basis of the institutional role held within the society—in several cases the incumbent president—combined with recognized clinical and research experience in cancer pain management and a documented publication record in the field. The composition of the Committee, therefore, reflects the six disciplinary constituencies involved in cancer pain care in Italy, together with the patient advocacy organization that convened the initiative, rather than the preferences of any individual investigator.

A total of 66 Italian clinicians were invited to participate as Delphi panelists. Candidates were nominated by Steering Committee members on the basis of direct knowledge of their clinical activity and of their demonstrated engagement in research and quality-improvement initiatives in the field of pain. To be eligible, a nominee had to satisfy three requirements: (i) specialist qualification in medical oncology, radiation oncology, pain medicine, or palliative care; (ii) current, active involvement in the clinical management of patients with cancer pain within the Italian NHS; and (iii) consent to participate. No exclusion criteria were applied beyond failure to meet these requirements. No numerical quota was pre-specified for any specialty or geographical area, although the Steering Committee sought to ensure that all four specialties and all major geographical macro-areas of the country were represented among those invited. Nominees received a single invitation by email, containing a description of the initiative and the survey link.

The panel was deliberately restricted to physicians. The statements submitted for rating concern medical decision points—the initiation and titration of analgesic therapy, the definition of therapeutic failure, the indication for and timing of specialist referral—for which clinical responsibility rests with the treating physician, and the pathway was designed to specify the division of clinical responsibility among medical specialties. This is a deliberate delimitation of scope rather than a judgement on the contribution of other professionals to cancer pain care; its implications, and the case for a broader multiprofessional panel in future iterations, are discussed in Section 4.

### 2.4. Delphi Process

The survey was administered online via SurveyMonkey and was open from 1 October to 31 December 2025. Reminders were sent to non-responders every 10 days for the duration of the round. Provision had been made for two voting rounds; in the event, only one proved necessary (see Section 3). Panelists rated their agreement with each statement using a 5-point Likert scale (1 = total disagreement, 5 = total agreement). Each item also allowed open-ended qualitative comments, which were collected and would have informed the reformulation of any statement submitted to a second round.

All responses were anonymous. Anonymity is an intrinsic component of the Delphi method rather than a procedural preference: panelists could not see one another’s ratings or identities, and the Steering Committee received only aggregate data, with individual responses accessible to no member of the research team in identifiable form. This design limits the influence of professional hierarchy, specialty allegiance and conformity pressure on individual judgements, which is the principal methodological advantage of the Delphi technique over consensus conferences and nominal group techniques [13,14,17]. Panelists were free to skip any individual item without abandoning the survey; consequently, the denominator for each statement is the number of panelists who rated that specific item, and this number is reported separately for every statement in Table 1.

Consensus was defined a priori as ≥75% of respondents scoring 4 or 5 for a given statement, in line with the threshold most frequently adopted in the published Delphi literature [14]. Statistical analysis comprised the calculation of response frequency distributions and the determination of consensus attainment for each statement. Percentages are reported to one decimal place throughout, and are calculated on the number of panelists rating each individual item.

## 3. Results

Of the 66 clinicians invited to participate, 56 completed the survey, corresponding to a response rate of 84.8%. Ten invitees returned no response; no reason for non-participation was communicated to the Steering Committee, and no further information on non-responders is available. Respondents represented a broad multidisciplinary spectrum: pain therapists (26.8%, *n* = 15), radiation oncologists (25.0%, *n* = 14), palliative care physicians (25.0%, *n* = 14), and medical oncologists (23.2%, *n* = 13).

Because panelists were permitted to skip individual items, the number of respondents rating each statement ranged from 53 to 54, corresponding to two or three unrated items per statement; all 56 participants provided professional data and rated at least one statement. The denominator applicable to each statement is reported in Table 1 and is the basis of all percentages given below.

Consensus (defined as ≥75% of respondents scoring 4 or 5 on the 5-point Likert scale) was reached for all 15 statements in the first round (Table 1). Agreement rates ranged from 77.8% (Statement 5) to 100% (Statements 4 and 10). Given that all statements met the pre-defined consensus threshold in the first round, a second Delphi round was not conducted. However, the panelists’ qualitative comments were reviewed by the Steering Committee, and minor refinements were applied to the wording of selected statements to improve clarity and precision, without altering their substantial content.

For each of the 15 statements, the table reports the number of respondents who rated that item, the percentage distribution of responses across the 5-point Likert scale (1 = total disagreement, 5 = total agreement), and the combined percentage of responses scoring 4 or 5 (consensus rate). Consensus was defined a priori as ≥75% of respondents rating a statement 4 or 5. All 15 statements reached consensus in the first round. Panelists were permitted to skip individual items; percentages are therefore calculated on the number of panelists who rated each specific statement (*n*), not on the 56 panelists who took part overall. Percentages are given to one decimal place and may not sum to exactly 100.0 owing to rounding.

Statements 1 through 3 addressed the role of the clinical reference physician in initiating and coordinating pain management, achieving consensus of 98.1%, 98.1%, and 96.3%, respectively. Statement 4, concerning early post-initiation monitoring within 3–7 days, reached unanimous consensus (100%). Statement 5, which proposed a 30% reduction in pain intensity at 7 days as the threshold for defining treatment inefficacy, achieved the lowest—though still qualifying—consensus rate of 77.8%. Statements 6 through 11 addressed indications for specialist referral and multidisciplinary collaboration, with agreement ranging from 81.5% (Statement 6) to 100% (Statement 10). Statements 12 through 15, focusing on standardized clinical documentation and the content of the pain assessment form, all achieved a consensus of 96% or higher (range 96.2–98.1%).

Free-text comments were submitted for every statement. The majority endorsed the statement in question while proposing clarifications of wording or of scope: recurring themes were the need to specify which specialist should be involved in particular clinical situations, the organizational preconditions required for systematic early monitoring, and the applicability of fixed numerical thresholds and time intervals to individual patients. The most substantial body of comment concerned Statement 5 and is examined in Section 4.

## 4. Discussion

The present Delphi consensus gives operational form to the mandate established by the national multidisciplinary roundtable held in Rome on 28 March 2025 [8]. That meeting identified four major systemic gaps in Italian cancer pain management: insufficient education and training, fragmentation of care pathways, lack of clarity regarding professional roles, and difficulties in implementing shared diagnostic and therapeutic pathways (Percorsi Diagnostico-Terapeutici Assistenziali, PDTAs). The derivation of those four gaps—from the clinical experience of the participants, the available national survey data, and the documented shortfall in the implementation of Law 38/2010—is described in Section 2.2 and reported in full in the proceedings of the roundtable [8]. The present study addresses each of these gaps through a structured, evidence-based, and expert-validated consensus process. In our view, the main strength of this Delphi consensus lies in the high number of respondents, with 85% of invited panelists that completed the survey, a response rate that compares favorably with benchmarks for Delphi studies in clinical medicine [13]. In a domain where engagement with structured quality-improvement initiatives has historically been uneven, this level of participation reflects a genuine and broad interest among the Italian clinical community. It reinforces the representativeness of the consensus achieved.

**Figure 1:**
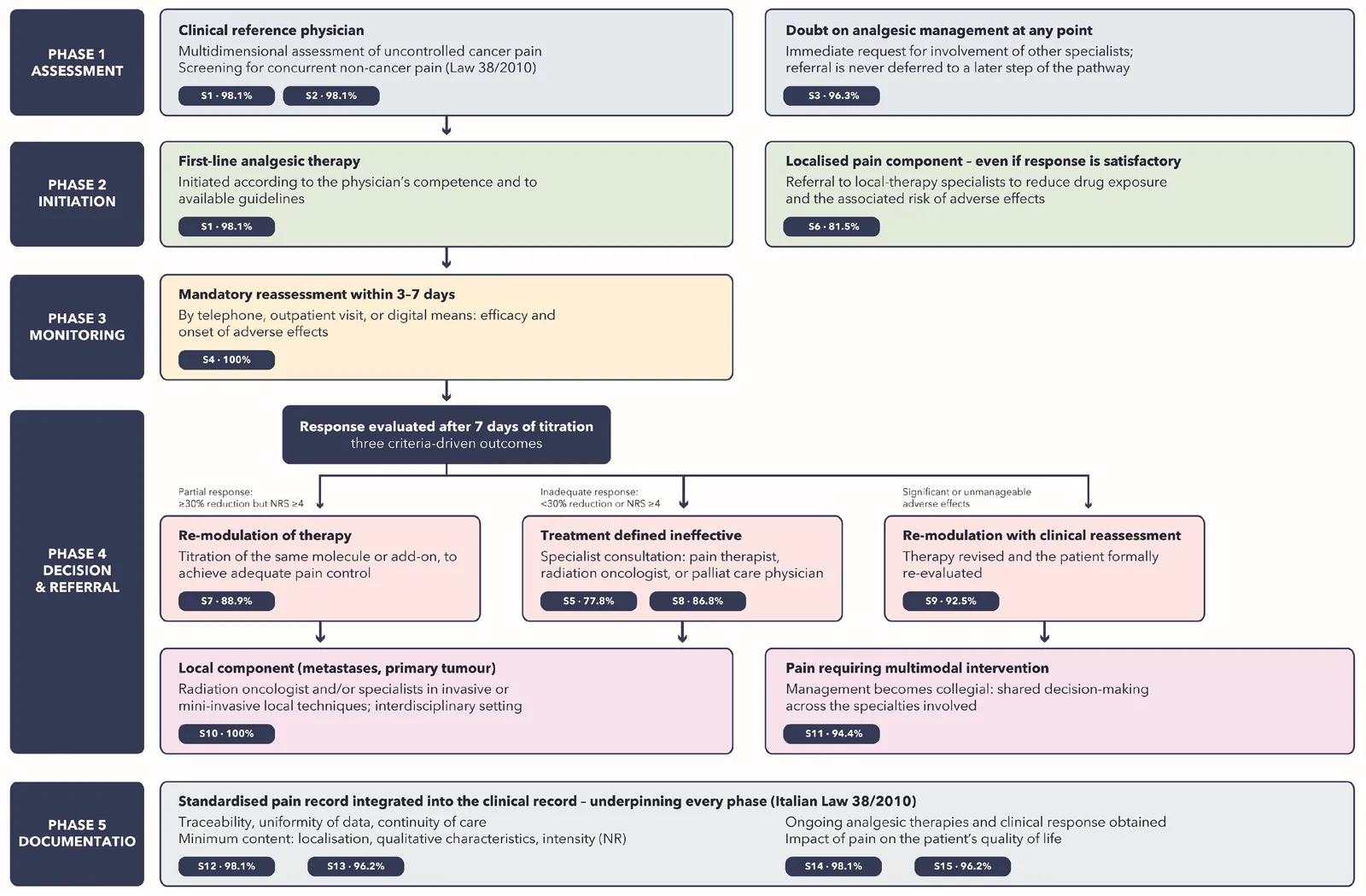
Delphi-validated multidisciplinary pathway for cancer pain management, organized in five phases derived from the 15 consensus statements (Table 1, S1–S15): assessment, therapy initiation, early monitoring at 3–7 days, a decision branch evaluating response after 7 days of titration, and criteria-driven specialist referral. Standardized pain documentation in compliance with Italian Law 38/2010 underpins all phases. Percentages are the proportion of panelists rating each statement 4 or 5 (consensus threshold ≥ 75%). NRS, Numerical Rating Scale.

A scheme of the pathway is provided in Figure 1. The pathway begins with the medical oncologist—or, more broadly, another specialist acting as the clinical reference physician—as the primary responsible actor for pain assessment and first-line analgesic-based pain management. Statements 1 and 2, both achieving 98.1% consensus, establish that the initial evaluation of the oncological patient with uncontrolled pain must include a multidimensional pain assessment and should also screen for the possible presence of non-oncological pain, consistent with Law 38/2010. Statement 3 (96.3%) further affirms that when doubts arise regarding the management of analgesic therapy, the clinical reference physician must seek promptly for other specialists’ involvement. This foundational triad of statements reflects a deliberate attempt to consolidate first-line pain management as a core competence of the clinical reference physician, rather than as a task to be delegated at the outset. Far from diminishing the role of the treating oncologist, this expands it: the physician who assesses pain multidimensionally, initiates analgesia and recognizes the point at which a colleague must be involved is exercising a broader clinical competence, not a narrower one. The intent is to make adequate first-line pain management an expected and attainable part of routine oncological practice, thereby addressing the educational gap identified by the roundtable and consistent with evidence that only a minority of Italian oncologists currently assess pain at every clinical visit [9,10].

Once analgesic therapy is initiated, the pathway establishes strict monitoring timelines. Statement 4—a statement achieving 100% consensus across all respondents—affirms that follow-up on an outpatient basis (by telephone, outpatient consultation, or digital means) within the first 3–7 days is essential to verify efficacy and/or the onset of adverse effects. This unanimous endorsement reflects a convergent clinical view that early monitoring is not optional but essential for adequate pain management. The therapeutic target is a reduction of at least 30% in NRS score within 7 days of titration initiation; failure to reach this threshold defines treatment inefficacy (Statement 5, 77.8%). In cases of partial response, defined as a reduction in NRS of at least 30% with a residual NRS score of 4 or higher, treatment re-modulation is indicated, either through dose titration of the same agent or by adding adjunctive therapy (Statement 7, 88.9%). These sequential thresholds provide the clinical reference physician with an objective, time-anchored decision framework that mitigates the risk of anchoring bias and therapeutic inertia, two well-documented contributors to analgesic undertreatment in oncology [3,4].

Statement 5 recorded the lowest consensus of the exercise (77.8%) and attracted the largest volume of qualitative comment; it therefore merits explicit discussion. The ≥30% criterion is not arbitrary. In a pooled analysis of chronic pain trials, a reduction of approximately 30% on the 11-point NRS corresponded to the smallest change that patients themselves reported as meaningful improvement, and this figure has since been widely adopted as the standard definition of analgesic response, including in international guidance on cancer pain [18,19]. Several panelists nonetheless contested its use as a categorical definition of treatment failure, and three objections recurred. First, a fixed percentage may be insensitive to baseline intensity: a fall from NRS 10 to NRS 7 satisfies the criterion while leaving the patient in severe pain. Second, several panelists argued that the therapeutic target should be the patient’s personalized pain goal rather than a population-derived cut-off—a position supported by evidence that the large majority of patients with cancer can state a desired level of pain relief, that this level is stable over time, and that goal attainment classifies response differently from the ≥30% criterion [20]. Third, panelists observed that a 7-day horizon may be too long in advanced disease, and that response should be interpreted alongside function, sedation and quality of life rather than in isolation—a patient rendered pain-free but sedated and unable to interact with caregivers should not be counted as a therapeutic success. The Steering Committee regards these objections as complementary to the statement rather than incompatible with it. The ≥30%/7-day criterion is intended as a minimum procedural safeguard—a defined point at which the pathway obliges the clinician to act rather than to wait—and not as a definition of adequate analgesia; Statement 7 makes this explicit by requiring re-modulation whenever residual NRS is ≥4 even where the 30% threshold has been met. The statement exceeded the pre-specified consensus threshold and therefore stands, but the distribution of responses—13.0% neutral and 9.3% in disagreement, the highest of any statement—is reported transparently in Table 1 and identifies this criterion as the element of the pathway most in need of prospective evaluation, and the one most likely to require adaptation to the individual patient.

A defining feature of the validated pathway is its non-sequential, criteria-driven approach to another specialist referral—a deliberate departure from the hierarchical cascade that has historically delayed access to pain specialists and palliative care [18,21]. If pain is not controlled despite adequate titration within 7 days (NRS ≥ 4 or intensity reduction < 30%), specialist consultation with a pain therapist, radiation oncologist, or palliative care specialist is indicated (Statement 8, 86.8%). Equally, when adverse effects are important or difficult to manage with the current therapy, specialist reassessment must be sought (Statement 9, 92.5%). Crucially, whenever pain has a local component—such as that arising from bone metastases or direct tumor involvement—prompt consultation with a radiation oncologist and/or specialists in minimally invasive local techniques is indicated, preferably within an interdisciplinary setting (Statement 10, 100%). Whenever pain characteristics require multimodal intervention, its management must become collegial (Statement 11, 94.4%). Taken together, these statements establish a model in which any member of the team can trigger specialist involvement on the basis of objective clinical parameters, rather than only after a fixed sequence of prior steps has been exhausted.

The evidence base underlying each specialist’s contribution to this framework is well established. Palliative radiotherapy achieves clinically meaningful pain relief in approximately 60–75% of patients with bone metastases and other localized pain syndromes [22,23,24]. Moreover, a recent meta-analysis revealed that the majority (85%) of studies investigating the efficacy of radiofrequency ablation of painful spinal metastasis reported highly effective pain management (≥4-point pain score reduction) [25]. Statement 6 (81.5%) extends this logic further: even when pharmacological therapy is producing a satisfactory response, referral to local therapy specialists is appropriate if a localized pain component is present, in order to reduce overall drug exposure and the associated risk of adverse effects. Pain therapy specialists offer access to interventional approaches—nerve blocks and/or ablation, neuromodulation, intrathecal drug delivery—with demonstrated efficacy in neuropathic and refractory pain states [1]. Palliative care integration from early in the disease trajectory has been associated with improved symptom control, reduced emergency admissions, and better alignment with patient goals [6,15]. The interdisciplinary management model endorsed in Statement 11 institutionalizes shared decision-making without displacing individual specialist authority.

Although an improvement has been observed in the last decades, a persistent structural barrier to appropriate pharmacological management in Italy is the modest use of opioids—prescribing hesitancy rooted in knowledge gaps, regulatory concerns, and sociocultural attitudes—which leads to systematic underutilization of opioid analgesics even in patients with moderate-to-severe cancer pain [26]. The decision thresholds validated in Statements 1–9 are designed in part to counteract this barrier: by providing objective, consensus-backed criteria for analgesic initiation, titration, and escalation, the pathway makes guideline-concordant opioid prescribing a structurally supported clinical behavior rather than a matter of individual discretion. Non-pharmacological and integrative approaches are equally recognized: Statement 6 affirms the value of local therapies in reducing pharmacological burden, and the broader literature supports the integration of psychological, rehabilitative, and complementary interventions within a multimodal pain management strategy [12,27].

Among the most unambiguous outcomes of the Delphi process is the near-unanimous endorsement of a standardized, integrated pain record as a structural component of the oncological clinical record. All four statements pertaining to the standardized pain documentation record—Statements 12 through 15—achieved consensus rates between 96.2% and 98.1%. Statement 12 (98.1%) establishes that the clinical record should incorporate a standardized form ensuring symptom traceability, data uniformity, and care continuity, in compliance with Law 38/2010. Statement 13 (96.2%) defines the mandatory minimum content: pain localization, qualitative characteristics, and intensity measured with a validated scale such as the NRS. Statement 14 (98.1%) requires documentation of analgesic therapies in progress and their clinical outcomes. Statement 15 (96.2%) recommends inclusion of an assessment of pain’s impact on the patient’s quality of life, coherently with the increasing recognition of the importance of patient-reported outcomes in the continuum of care for patients with cancer [28]. Italy’s Law 38/2010 has mandated systematic pain documentation since 2010, yet adherence has remained fragmented, particularly outside specialist centers [8,17]. In a previous Delphi consensus among Italian medical oncology specialization school directors, a form was validated, which provides a concrete, widely endorsed instrument to close this implementation gap and simultaneously enables longitudinal symptom traceability and inter-center care medical continuity [17].

The recommendation of including quality of life as an assessment dimension (Statement 15) also reflects the fundamental inseparability of pain and QoL across all phases of the cancer care continuum. Pain and fear of recurrence represent the two Health-related-QoL subdomains most consistently rated as important by patients at all stages of disease [16]. Uncontrolled pain is associated with social isolation, loss of daily functioning, psychological distress, financial toxicity, and—in patients with advanced cancer—shortened survival [15,21,29].

A legitimate question is what this consensus adds to existing international recommendations. The ESMO clinical practice guidelines and the WHO guidance provide detailed, evidence-graded direction on the pharmacological and radiotherapeutic treatment of cancer pain: which analgesic to initiate, how to titrate it, how to manage breakthrough pain and adverse effects, and when radiotherapy is indicated [7,18]. What they do not provide—because it was never their object—is an operational description of the division of clinical labor: who holds responsibility for pain at each point of the oncological pathway, on what criteria and within what interval that responsibility should be shared with another specialist, and what must be recorded so that the transition is traceable. That is the space the present consensus occupies, and its distinctive elements are three. First, referral is criteria-driven rather than sequential: any objective trigger—inadequate response at 7 days, unmanageable toxicity, the presence of a local pain component—authorizes direct specialist involvement, without the hierarchical cascade implicit in a stepwise model, and any member of the team may activate it. Second, the triggers are time-anchored, converting the injunction to “refer when appropriate” into a defined interval that can be audited. Third, the pathway binds these decisions to a standardized documentation instrument, giving the requirements of Law 38/2010 a concrete clinical form [17]. The statements are therefore not an alternative to ESMO or WHO guidance but an organizational layer built upon it, adapted to the specialty structure and the regulatory framework of the Italian National Health System.

The conditions for implementation are, however, uneven. Successive reports to the Italian Parliament on the enactment of Law 38/2010 have documented that organizational models for palliative care and pain therapy networks are not uniform across the country, and that patient pathways differ substantially between regions [30]; national survey data show the same heterogeneity at the level of clinical behavior and opioid prescribing [9,26]. Several statements presuppose resources that are not universally available. Statement 4 assumes the organizational capacity to contact a patient within 3–7 days of initiating therapy—a point raised explicitly by panelists, who noted that structured follow-up of this kind requires protected time and dedicated operating procedures rather than individual goodwill. Statements 6 and 10 assume timely access to radiation oncology and to interventional pain techniques, which is unevenly distributed geographically. Statements 12–15 assume an electronic clinical record capable of accommodating a structured pain form, which panelists reported is frequently not the case in general medical and oncology wards, where such data must be entered manually. Where these preconditions are absent, the pathway should be read as defining the standard to be worked towards, and as an argument for the organizational investment required to reach it, rather than as an immediately achievable protocol. The same caution applies a fortiori to health systems with more constrained resources, in which the availability of opioid analgesics may itself be the binding constraint [6,7].

Two constituencies were represented in this process only indirectly. Neither patients nor caregivers took part in the voting panel, and no formal patient-reported input was collected. This is a substantive rather than a formal limitation: several of the statements—the definition of an adequate analgesic response, the acceptable trade-off between pain relief and adverse effects, the choice of what should be recorded about quality of life—turn on judgements for which patient and caregiver perspectives are not interchangeable with clinical ones, as the debate on Statement 5 illustrates. Delphi methodology accommodates mixed panels of professionals and lay experts, and recent Italian experience has shown that patients, caregivers and their representatives can be integrated into structured consensus processes in oncology [27]; future iterations of this pathway should do so. Similarly, and by deliberate design, the panel did not include oncology nurses, clinical pharmacists, psychologists, or rehabilitation specialists, since the statements address decisions for which the physician carries clinical and medico-legal responsibility; the present document should accordingly be read as expressing the multidisciplinary medical viewpoint. It is nonetheless evident that implementation will depend substantially on these professionals—nurses perform the greater part of in-hospital pain monitoring and documentation, clinical pharmacists influence opioid availability and prescribing appropriateness, and psychological and rehabilitative interventions are integral to multimodal management. Their inclusion in a subsequent, implementation-oriented consensus is a natural extension of this work, and one that the Steering Committee regards as necessary.

From a methodological standpoint, the study presents several strengths. Adherence to CREDES guidelines ensured procedural rigor and reporting transparency [13]. The steering committee comprised representatives of six major Italian clinical societies, and the Delphi panel included 56 multidisciplinary clinicians with broad geographic representation. Consensus was defined at a pre-specified threshold of ≥75% of respondents rating a statement 4 or 5 on a 5-point Likert scale. It was reached for all 15 content statements, with multiple items approaching or attaining 100% agreement. Anonymous voting and open qualitative comment collection in each round further reinforced validity. Nonetheless, several limitations must be acknowledged. The statements were informed by a targeted rather than a systematic literature search, and no formal grading of the level of evidence supporting each statement was performed; the statements consequently represent expert consensus informed by the literature, not evidence-based recommendations in the technical sense. No pilot administration preceded the voting round, so the psychometric properties of the instrument were not formally assessed, although all statements were reviewed for clarity and face validity by the full Steering Committee. Because the pre-specified threshold was met by every statement at the first round, the process terminated after a single round: this is a legitimate consequence of the stopping rule, but it means that panelists had no opportunity to revise their ratings in the light of controlled feedback, and that the stability of consensus across rounds could not be assessed. Panelists were also permitted to skip individual items, and 2–3 did so for each statement; while this preserves the validity of the denominators reported in Table 1, the ratings of those panelists are unknown. The pathway was developed specifically within the Italian NHS framework and reflects its regulatory context, regional infrastructure, and specialty organization; adaptation to other healthcare systems would require contextual revision. Neither patients nor caregivers were directly represented in the panel, and the panel was restricted to physicians, as discussed above. No pediatric component was included, which would require separate development. Finally, and importantly, the present study produces a consensus-validated framework; its clinical impact on patient outcomes in real-world practice remains to be evaluated through prospective implementation research. The present consensus is therefore best understood as a necessary but not sufficient step in a longer continuum: from the national roundtable that identified the gaps, to this Delphi that translates them into actionable clinical statements, to the implementation and evaluation work that must follow.

## 5. Conclusions

This multisociety Delphi process produced a multidisciplinary clinical pathway for cancer pain management in the Italian National Health System, with all 15 statements reaching consensus at the first round (77.8–100%). The pathway assigns first-line responsibility for multidimensional pain assessment and analgesic initiation to the clinical reference physician, defines objective and time-anchored criteria for judging therapeutic response, replaces the sequential referral cascade with criteria-driven access to pain medicine, radiation oncology and palliative care, and specifies the minimum content of a standardized pain record consistent with Law 38/2010. In doing so it addresses, in operational terms, the four systemic gaps identified at the 2025 national roundtable.

Consensus, however, is a starting point rather than an endpoint. The value of these statements will be determined by whether they change clinical behavior, and that question is empirical. We therefore regard prospective evaluation as the necessary next step, with implementation outcomes specified in advance: the proportion of oncological visits at which pain is assessed and documented with a validated instrument; the interval between the initiation of analgesic therapy and the first reassessment; the proportion of patients with uncontrolled pain referred to a pain specialist, radiation oncologist or palliative care physician within the defined interval; and, ultimately, pain intensity and quality of life reported by patients themselves. A subsequent iteration of the pathway should also broaden the panel to include patients, caregivers, nurses, pharmacists and rehabilitation professionals, whose contribution to implementation is decisive and whose perspectives the present exercise did not capture.

## Figures and Tables

**Table 1:** Results of the first-round Delphi survey on cancer pain management (*n* = 56 respondents).

No.	Statement	*n*	1	2	3	4	5	Consensus (4 + 5)
Domain 1—Pain Assessment								
1	The first phase of care for cancer patients with uncontrolled pain (absence of therapy or inadequate therapy—in terms of efficacy or side effects) requires the referent physician to perform a multidimensional pain assessment and to initiate first-line analgesic medical therapy, according to their competence and available guidelines.	54	0.0	0.0	1.9	11.1	87.0	98.1
2	The clinical referent of the cancer patient should also assess the possible presence of uncontrolled non-cancer pain, in line with Italian Law 38/2010, and encourage the patient to initiate an appropriate management pathway with the relevant specialists.	53	0.0	0.0	1.9	18.9	79.2	98.1
3	If the clinical referent has any doubts regarding pain therapy management, they must immediately request the involvement of other specialists.	54	0.0	0.0	3.7	20.4	75.9	96.3
Domain 2—Treatment Initiation and Monitoring								
4	After initiating analgesic therapy, monitoring (by phone, outpatient visit, or digital means) within the first 3–7 days is essential to assess efficacy and the occurrence of any side effects.	54	0.0	0.0	0.0	13.0	87.0	100.0
5	If the initiated treatment does not achieve a reduction of at least 30% in pain intensity within 7 days of therapy titration, it should be considered ineffective.	54	1.9	7.4	13.0	44.4	33.3	77.8
6	Local therapy can also help minimise pharmacological intervention, to which adverse effects may be associated. Therefore, even in cases of satisfactory response to pharmacological therapy, in the presence of localised pain it is appropriate to refer the patient to local therapy specialists, in order to reduce exposure to potentially adverse-effect-associated drugs.	54	1.9	1.9	14.8	29.6	51.9	81.5
Domain 3—Escalation and Multimodal Management								
7	In the case of partial response to analgesic treatment, defined as a reduction ≥30% in pain intensity but with NRS ≥4, re-modulation of therapy (titration of the same molecule or add-on) is indicated to achieve adequate pain control.	54	0.0	0.0	11.1	31.5	57.4	88.9
8	If, despite an adequate titration process within 7 days, pain is not controlled (NRS ≥ 4 or intensity reduction < 30%), a specialist consultation is indicated (Pain Therapist, Radiation Oncologist, Palliativist).	53	1.9	1.9	9.4	15.1	71.7	86.8
9	In the presence of significant or difficult-to-manage adverse effects with ongoing therapy, re-modulation of therapy with clinical reassessment is required.	53	1.9	1.9	3.8	30.2	62.3	92.5
10	In the presence of pain with a local component (e.g., from metastases or primary tumour), consultation with a Radiation Oncologist and/or specialists in local invasive or mini-invasive techniques (Pain Therapist, Orthopaedic Surgeon, Neurosurgeon, Interventional Radiologist) is indicated; when possible, an interdisciplinary approach is recommended.	53	0.0	0.0	0.0	20.8	79.2	100.0
11	Whenever the characteristics of pain require multimodal intervention, management must become collegial.	54	0.0	0.0	5.6	16.7	77.8	94.4
Domain 4—Documentation and Clinical Record								
12	It is desirable that the clinical record integrates a standardised form for pain assessment and monitoring, to ensure symptom traceability, uniformity of collected data, and care continuity, in line with Italian Law 38/2010.	53	0.0	1.9	0.0	7.5	90.6	98.1
13	The clinical pain assessment form must include at least pain localisation, qualitative characteristics, and intensity, measured with validated scales such as the NRS.	53	0.0	0.0	3.8	7.5	88.7	96.2
14	The clinical form must report the ongoing analgesic therapies and the results obtained in terms of clinical response.	53	0.0	0.0	1.9	3.8	94.3	98.1
15	In the clinical form, it is recommended to include an assessment of the impact of pain on the patient’s quality of life.	53	0.0	0.0	3.8	15.1	81.1	96.2

## Data Availability

The aggregated, anonymized data supporting the findings of this study are available from the corresponding author upon reasonable request. Individual panelist responses are not publicly available in order to preserve participant confidentiality.

## Appendix A

**The Cancer Pain Management in Italy Working Group:** Dario Alicino, Fabio Arcidiacono, Grazia Armento, Alessandra Marina Bertola, Claudia Bighin, Chiara Maria Giovanna Broglia, Filippo Canzani, Simona Carnio, Angelo Antonio Carrideo, Cinzia Casini, Benedetta Chellini, Rosario Chianese, Elisabetta Chine, Antonella Ciabattoni, Rita D’Urso, Marta De Angelis, Italo Dell’Oca, Nicoletta Di Francesco, Rossella Di Franco, Costanza Maria Donati, Giacomo Ferrantelli, Luigi Formisano, Flavio Fusco, Marta Rita Gatta Micheletti, Patrizia Giardina, Raffaele Giusti, Nicola Alessandro Iacovelli, Massimo Innamorato, Gaetano Lanzetta, Marco Vincenzo Lenti, Sergio Mameli, Stefania Manfrida, Manuela Margaritelli, Franco Marinangeli, Renata Marinello, Francesca Maurizi, Marco Mercieri, Federica Melazzini, Luca Miceli, Alessio G. Morganti, Andrea Pession, Donato Pezzulla, Tania Piccione, Antonio Portoriero, Marianna Rondini, Romina Rossi, Rodolfo Sacco, Daniele Santini, Emiliano Tamburini, Davide Tassinari, Francesca Tortoreto, Marcello Tiseo, Anna Tiziano, Ciro Visconti, Renato Vellucci and Vittorina Zagonel.
